# Measles serostatus among health care workers in a university clinic in Hungary in 2024

**DOI:** 10.1016/j.pmedr.2026.103496

**Published:** 2026-05-08

**Authors:** Bálint Timmer, Benigna Balázs, Ákos Boros, Antal Tibold, Andor Sebestyén, Gábor Reuter

**Affiliations:** a Department of Medical Microbiology, Medical School, University of Pécs, Pécs, Hungary; b Centre for Occupational Medicine, Medical School, University of Pécs, Pécs, Hungary; c Clinical Center, Medical School, University of Pécs, Pécs, Hungary

**Keywords:** Measles, Morbilli, Healthcare workers, Vaccine-preventable diseases, Healthcare-associated infections, Seroprevalence, Immunity gaps, MMR vaccination, Hungary

## Abstract

**Objective:**

Healthcare workers (HCWs) face elevated risk of measles exposure and may facilitate nosocomial spread. Despite early (1969) national vaccine introduction and high (>99%) coverage, sporadic cases and small outbreaks tend to occur in Hungary pointing to possible immunity gaps. We evaluated quantitative measles IgG levels among HCWs in a Hungarian university clinic to assess immunity against measles morbillivirus.

**Methods:**

Between July and November 2024, sera from 2541 HCWs were tested by quantitative ELISA. Antibody titres were categorized into four groups, with >200 mIU/mL considered protective immunity. IgG levels were analyzed by age, sex, and vaccination schedules.

**Results:**

Overall, 78.83% had protective antibody levels, while 21.17% were below the protective threshold, including 13.81% with IgG <150 mIU/mL. Unvaccinated group (born before 1968) provided the highest antibody titres exceeding vaccinated groups. Significant differences in serum titres detected between individuals received different vaccine regiments by age groups. Newer combinations, such as MMR-II/Priorix, elicited higher antibody levels compared to old combinations. HCWs aged 39–50 years showed the highest (32–38%) susceptibility.

**Conclusions:**

A substantial proportion of HCWs have low level of measles antibodies, especially in specific age cohorts. Targeted screening and revaccination are recommended to close immunity gaps and prevent nosocomial transmission.

## Highlights

• 78.83% of healthcare workers had protective measles IgG, 21.17% remained susceptible.
• The highest susceptibility observed between 39 and 50 years of age.
• Natural infection induced the highest measles IgG antibody titres.
• Antibody titres differed significantly across different vaccination statuses.
• Screening and targeted revaccination needed to close immunity gaps.

## 1 Introduction

Measles remains one of the most contagious infectious diseases, with serious implications for both public health and healthcare workers (HCWs). Despite the global success of vaccination programs, measles infections continue to occur, particularly in settings with suboptimal vaccination coverage (van den Hof et al., 2001). HCWs, as frontline responders in managing infectious diseases, are at increased risk of exposure and transmission, both to themselves and their patients. Studies have reported varying rates of measles susceptibility among HCWs, posing an ongoing public health concern (Bianchini et al., 2022; Lengyel et al., 2018). However, susceptibility rates differ between countries and regions, while available data remain incomplete.

The consequences of measles susceptibility among HCWs extend beyond individual health risks, as susceptible HCWs may contribute to nosocomial transmission (Martin et al., 2012). Two doses of the mumps–measles–rubella (MMR) vaccine provide approximately 97% protection against measles, while a single dose offers about 93–95% effectiveness (Di Pietrantonj et al., 2021). Vaccination coverage of more than 92–95% is required to achieve herd immunity and protect susceptible individuals (Anderson and May, 1990). However, measles vaccination coverage remains below 95% in Europe (Simone et al., 2012; European Centre for Disease Prevention and Control (ECDC), 2024). In line with the World Health Organization (WHO), the Centers for Disease Control and Prevention (CDC) recommend that HCWs should have documented evidence of immunity through laboratory confirmation or vaccination records (World Health Organization (WHO), 2017; Centers for Disease Control and Prevention (CDC), 2025).

Assessment of measles immunity among HCWs is an important component of healthcare-associated infection control. Immunity levels among HCWs reflect not only individual protection but also protection of vulnerable patient groups, including unvaccinated infants, immunocompromised individuals or pregnant women.

There are significant differences between and within countries of the introduction time of measles vaccination, the type of vaccine used, and the number of doses administered. In Hungary, several changes have occurred in the national vaccination program over time (Rigó et al., 2020; Ferenczi et al., 2025). A single-dose monovalent measles vaccine (live, attenuated Leningrad-16 strain) was introduced in 1969 as a campaign for children aged 9–27 months. Measles vaccination became mandatory in 1974. Between 1984 and 1989, a single-dose monovalent vaccine was administered at 15 months of age. Between 1989 and 1991, this was replaced by a two-dose monovalent schedule (Rimevax) at 15 months and 11 years. Since 1991, a two-dose trivalent MMR vaccine (since 2019 MMRV) has been administered at 15 months and 11 years (Ferenczi et al., 2025; Böröcz et al., 2020; Szinger et al., 2024). Consequently, different age groups received different vaccines and schedules in Hungary, potentially leading to differences in serostatus.

Monitoring serological status is therefore important for maintaining immunity in healthcare settings (Lengyel et al., 2018). Since 2021, serological testing has been legally mandatory in Hungary for HCWs who cannot provide documentation of measles vaccination. If protective antibody levels are not detected, revaccination may be offered. Assessing seroepidemiology among HCWs can help identify immunity gaps and support measles elimination efforts (Szinger et al., 2024).

In this study, serum measles IgG antibody levels were measured using a quantitative ELISA method in 2541 HCWs at the Clinical Centre of the University of Pécs (Pécs, Hungary) to assess measles immunity status.

## 2 Materials and methods

### 2.1 Study design and population

Retrospective, serological screening study was performed, based on the Article 23/B (related to the measles serosurvey campaign among HCWs) of the NM Decree 18/1998 in Hungary. According to this, HCWs who presented documentation of their mandatory childhood measles vaccination did not have to undergo measles serological testing. The serum measles IgG antibody titres were measured in 2541 healthcare workers (physicians, nurses, laboratory workers) as a study pool who cannot (or does not want) provide documentation of measles vaccination (inclusion criteria). The Clinical Centre of University of Pécs represents the university clinic in Pécs and 5 surrounding town hospitals (Harkány, Komló, Mohács, Siklós, and Szigetvár) in Baranya County, Hungary.

### 2.2 Laboratory measures

Blood samples were collected between July 1, 2024 and November 8, 2024. Blood samples daily delivered to the laboratory on the day of the collection during the sample collection period. Serum was obtained from the blood samples immediately after arrival by centrifugation. The test was performed on the fully loaded ELISA plate within a maximum of 3–4 working days after the sample arrived. Serum samples were analyzed using the quantitative Measles Virus IgG ELISA kit (Institut Virion/Serion GmbH, Würzburg, Germany, Lot Nos.: EP0052, EP0166, EP0180), following the manufacturer's instructions. Antibody titres (mIU/mL) were calculated from absorbance values using the manufacturer's protocol, compared to the WHO international standard.

Based on the measles IgG titres (primary outcome variable), specimens were categorized into four groups: 0 mIU/ml, <150 mIU/ml, 150–200 mIU/mL, and > 200 mIU/mL based on the manufacturer's instructions. The measles IgG level > 200 mIU/mL defined as the threshold for an immunity status as “protective antibody level” according to the manufacturer's instructions which is also supported by international literature (Chen et al., 1990; Bolotin et al., 2020).

### 2.3 Statistical analysis

Age and sex data, as well as the results of laboratory tests, were collected from the MedBakter (Prolab Ltd.) medical system of the University of Pécs. Statistical analyses were conducted to assess differences in measles IgG antibody titres across age, sex and groups with different vaccination history. The specific vaccine groups were assigned only by the age (year of the birth) of participants (Supplementary Table 1). Personal health data about the measles infection recovery had not been available in the study population. However, the mandatory childhood measles vaccination at all times is homogeneous in the population, and exceptionally high, more than 99% in Hungary since 1974 (https://immunizationdata.who.int/dashboard/regions/european-region/HUN). Individuals born before the introduction of measles vaccination (<1968) who had protective antibody levels were considered naturally immunized. Individuals who received mandatory childhood measles vaccination (>1974) were classified as vaccinated, while those born before 1968 were considered unvaccinated due to the absence of childhood vaccination. Individuals born between 1968 and 1973 were classified as potentially vaccinated within the framework of the campaign measles vaccination program [group “Leningrad-16 1X (Campaign)”].

Shapiro–Wilk and Kolmogorov–Smirnov tests were used to assess the normality of age distributions and antibody titres. The Mann–Whitney *U* test was used to compare measles IgG titres between male and female participants. Kruskal–Wallis test was applied to compare antibody levels across vaccine groups and naturally immunized individuals. Post-hoc pairwise comparisons were performed using Dunn's test to evaluate differences between specific groups. Antibody titres were compared between individuals of different ages with Kruskal-Wallis test. Titers of different age groups were assessed using the Mann–Whitney *U* test. Differences in the proportion of susceptible individuals between groups of different vaccination status were analyzed using the Chi-square test. Statistical analyses and visualization were conducted in Python (version 3.13.2).

## 3 Results

A total of 2541 (50.79%, the total target workforce population consists of 5003 HCWs) individuals were included in the study; 1899 females (74.73%) and 642 males (25.27%) (Supplementary Fig. 5, Supplementary data 1–2, Supplementary data 7). The ages are ranged from 19 to 85 years, with the median age of 48 years (interquartile range, IQR: 37 to 55 years).

**Fig. 1:**
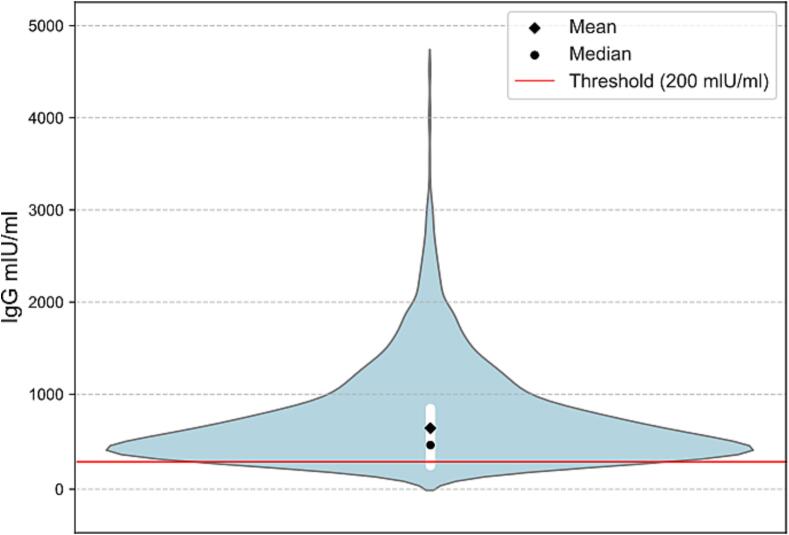
Violin plot showing the distribution of serum measles IgG antibody levels (mIU/mL) among healthcare workers (HCWs) in a Hungarian university clinic in 2024, which is not normally distributed (Supplementary Figs. 1–4).

**Fig. 2:**
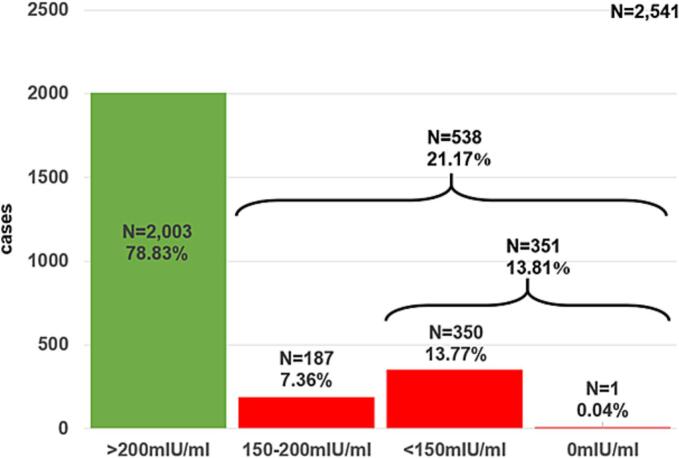
Column chart showing the incidence of HCWs (*N* = 2541) in a Hungarian university clinic in 2024 in the different categories based on the quantitative serum measles IgG antibody titer. Green column represents persons with protective measles immunity; red columns represent individuals with unprotective immunity. (For interpretation of the references to colour in this figure legend, the reader is referred to the web version of this article.)

Measles IgG antibody levels ranged from 0.0 mIU/ml to 4751.28 mIU/ml, with a median of 448.57 mIU/ml and a mean of 632.71 mIU/ml (Fig. 1). The distribution of measles IgG levels showed that 2003 (78.83%) individuals had titres above 200 mIU/ml indicating protective immunity, 187 (7.36%) had between 150 and 200 mIU/ml, while 351 (13.81%) had measles IgG titer below 150 mIU/ml including a person with 0 mIU/ml (Fig. 2). The 60-year-old HCW (born in 1964) with 0 mIU/ml measles IgG antibody titer had not received any vaccine against measles based on the medical history. Mann-Whitney *U* test comparing males and females showed no significant difference (*p* = 0.19) in measles immunity (Supplementary data 3–5).

**Fig. 3:**
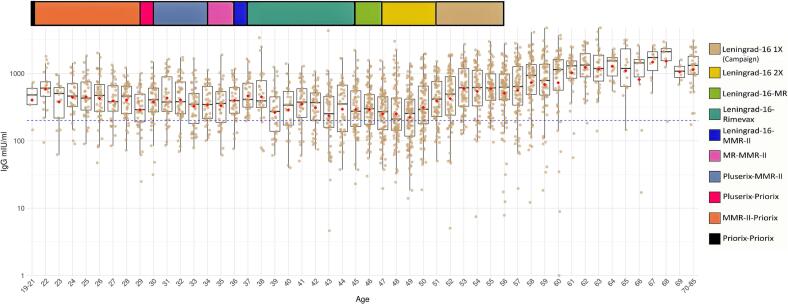
This log-normal scale dot plot shows the individual serum measles IgG levels (brown dots, N = 2541) by age groups among HCWs in a Hungarian university clinic in 2024. Each dot represents a titer value; blue dotted line indicates the susceptibility threshold (200 mIU/ml). Boxes ranging between the Q1 and Q3 quartiles, central lines indicating the median, whiskers ranging from the largest data point within 1.5 times the IQR above the upper quartile (Q3) to the smallest data point within 1.5 times below the Q1 quartile. Red diamonds showing the average values within datasets. Colored strips at the top shows the measles vaccine types and doses administered since 1969 in Hungary in aligned to the age groups. (For interpretation of the references to colour in this figure legend, the reader is referred to the web version of this article.)

**Fig. 4:**
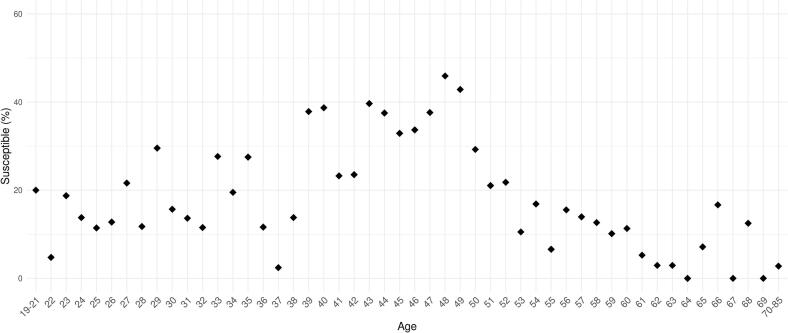
Dot plot demonstrating the proportion of susceptible individuals (<200 mIU/ml) for measles in percentage (y-axis) in case of each age group in years (x-axis) among HCWs in a Hungarian university clinic in 2024.

The measles IgG titer of each individual across different age groups is shown in Fig. 3. The lowest average titres were present in the age groups between 39 and 50 years, furthermore in these age groups were the highest rate of susceptible persons (32–38%, Fig. 3 and Fig. 4). This age group is the most represented in the total number of individuals (Supplementary Fig. 5). Antibody titres differed significantly across different age groups (*p* < 0.001). Furthermore, individuals aged 39–50 years showed significantly lower antibody titres compared to all other ages (p < 0.001). The proportion of susceptible individuals differed significantly between vaccine groups (χ^2^ = 171.5, degrees of freedom (df) = 8, p < 0.001) (Supplementary data 9).

**Fig. 5:**
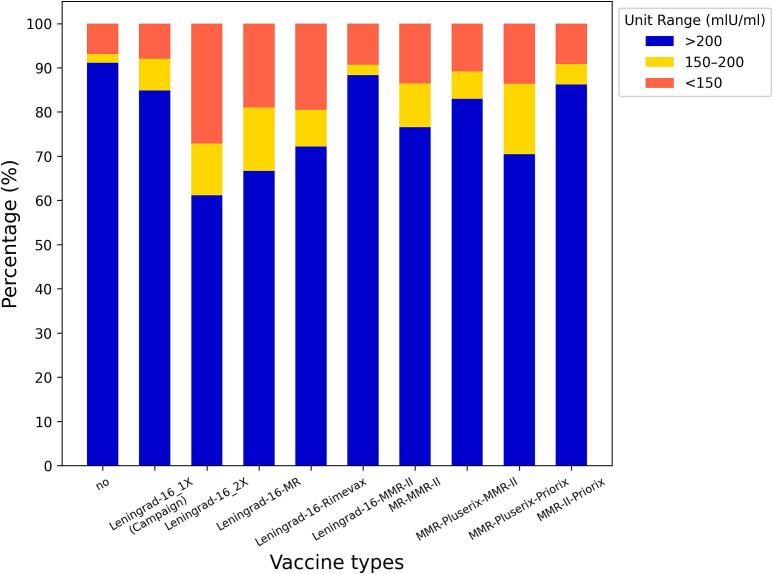
Bar chart representing the proportion of susceptible (<200 mIU/ml) and protected (>200 mIU/ml) population for measles in the respect of the type of measles vaccination among HCWs in a Hungarian university clinic in 2024.

Regarding measles vaccination status by age groups, 60.25% (*n* = 1531) were vaccinated (born later than 1974), 20.00% (*n* = 508) were unvaccinated (born before 1968), while 19.75% (*n* = 502) partially vaccinated (born between 1968 and 1973) as part of a measles vaccination campaign [Leningrad-16 1× (Campaign)]. Among mandatory vaccinated individuals, different vaccine formulations were administered: Leningrad-16, 2 shots (*n* = 417); Leningrad-16 and bivalent Measles-Rubella (*n* = 174); Leningrad-16 and Rimevax (*n* = 338); Leningrad-16 and MMR-II (*n* = 43); MR (measles-rubella) and MMR-II (*n* = 81); MMR-Pluserix and MMR-II (*n* = 194); MMR-Pluserix and Priorix (*n* = 44); MMR-II and Priorix (*n* = 239), and two shots of Priorix (n = 1) (Supplementary Table 1, Supplementary data 6). The highest proportion of individuals with IgG levels above 200 mIU/ml were among the naturally immunized (unvaccinated) group with 91.14%, the lowest among the individuals vaccinated with two shots of Leningrad-16 (61.15%). The MMR-II and Priorix combination, which was administered to people born between 1996 and 2004 resulted higher proportion of immunized individuals (86.25%) compared to the older combination (except for single shot of Leningrad-16 or Leningrad-16 with MMR-II booster) (Fig. 5). The highest IgG antibody titres were observed in the unvaccinated group (mean 1159.32 mIU/ml, median 1074.58 mIU/ml, IQR: 557.63–1581.33 mIU/ml). In case of partially and vaccinated groups, the highest antibody titres were detected in case of Leningrad-16 one shot with the median of 558.24 mIU/ml and mean of 690.54 mlU/ml (IQR: 295.94–983.43 mlU/ml), followed by MMR-II and Priorix combination (median: 463.92, mean: 532.18, IQR: 275.01–683.02 mlU/ml).

**Fig. 6:**
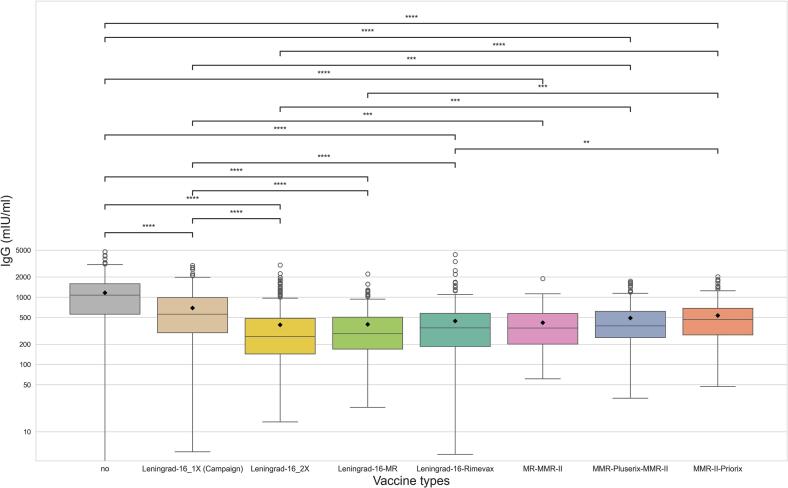
This log-normal scale Boxplot showing the range of serum measles IgG antibody levels in case of groups vaccinated with different type of vaccine formations, partially vaccinated [Leningrad-16 1× (Campaign)] or not vaccinated at all among HCWs in a Hungarian university clinic in 2024. Boxes ranging between the Q1 and Q3 quartiles, central lines indicating the median, whiskers ranging from the largest data point within 1.5 times the interquartile range (IQR) above the upper quartile (Q3) to the smallest data point within 1.5 times below the Q1 quartile. White circles displaying the outlier datapoints. Dunn's test annotations refer to the difference between datasets; *: 0.01 < *p* ≤ 0.05, **: 0.001 < *p* ≤ 0.01, ***: 0.0001 < *p* ≤ 0.001, ****: *p* ≤ 0.0001.

Kruskal-Wallis test revealed a significant difference in measles IgG antibody titres between vaccinated and unvaccinated individuals (*p* < 0.001), and across different vaccine types (p < 0.001, Supplementary data 8). Unvaccinated HCWs had significantly higher antibody titres than all vaccinated groups, regardless of the vaccine type (Fig. 6, Supplementary Table 2).

Post-hoc Dunn's test identified significant differences in measles IgG antibody levels between specific vaccine groups (Fig. 6). The naturally immunized unvaccinated group exhibited significantly higher measles IgG antibody titres compared to all vaccination combinations (*p* < 0.001 in each case). No significant differences were observed among the newer vaccination combinations (i.e., those not including Leningrad-16). Furthermore, in most cases, these newer combinations elicited significantly higher antibody levels compared to older regimens involving Leningrad-16 with various boosters. The MMR-II/Priorix combination resulted in significantly higher antibody levels compared to two doses of Leningrad-16, Leningrad-16/MR, and Leningrad-16/Rimevax combinations (Fig. 6, Supplementary Table 2).

## 4 Discussion

Measles serostatus among HCWs in a Hungarian university clinic was assessed in 2024 using quantitative measles IgG antibody titres. The majority of participants (78.83%) had titres above 200 mIU/mL, indicating protective immunity. However, 21.17% of HCWs had antibody titres below this threshold (<200 mIU/mL), including 13.81% with IgG levels below 150 mIU/mL, suggesting potential susceptibility to measles. Only one individual (0.04%) was seronegative (0 mIU/mL).

Two previous studies have evaluated measles seroepidemiology among HCWs in Hungary. The National Center for Public Health and Pharmacy reported protective antibody levels in 72.6% of HCWs based on 21,336 samples collected in 2017–2018 across six counties and two hospitals in Budapest with nationwide service obligations. The highest proportion of individuals with nonprotective antibody levels was observed in the 28–51-year age group [Rigó et al., 2020], consistent with our findings. Another study conducted at the Military Medical Centre in Budapest in 2017, including 2167 HCWs, reported higher overall seropositivity (90.5%), ranging from 86.2% to 99.1% depending on age group (Lengyel et al., 2018). The lowest seropositivity was found in the 35–45-year age group, which is in line with our observation in our 42–52-year age group seven years later. In that study, all HCWs were screened, not only those unable to present vaccination records, which may partly explain the differences. In Hungary, mandatory childhood vaccination coverage, including measles vaccination since 1974, has remained exceptionally high (e.g., the first/s MMR dose coverage was 99.8%/99.4% in 2024 (National Center for Public Health and Pharmacy, 2024a; https://www.antsz.hu/data/cms114786/OSAP_Vedooltas_2024_honlapra.pdf). All those studies, including ours, used the same ELISA kit, hence the results are objectively comparable.

When compared internationally, our results (78.83% seropositive) fall below those reported in Madrid, Spain, where de la Cuerda et al. found 89% of 2157 HCWs had protective antibody levels, with susceptibility associated with younger age and specific professional categories (de la Cuerda et al., 2024). Similarly, Coppeta et al. reported 85.2% seropositivity among 378 HCWs in Italy, with markedly higher immunity in individuals aged >35 years (98.3% vs. 79.2% in the 25–35 age group) (Coppeta et al., 2020). In our dataset, the 35–50-year age group showed higher susceptibility, which may be explained by differences in earlier vaccine formulations compared with those used in Western European countries. Nevertheless, our findings support international observations that herd immunity among HCWs remains incomplete.

Seroepidemiological studies from Central and Eastern Europe provide partially comparable results. In Poland, Smolinski et al. assessed measles IgG in 3000 individuals from the general population and reported seronegativity below 10% among those receiving one dose and below 6% among those receiving two doses of the Leningrad-16 vaccine, although the cohort was under 30 years of age and a different ELISA assay was used (Janaszek et al., 2000). In Romania, Stanescu et al. reported a nationwide seroprevalence of 77% without significant sex differences, while both seropositivity and antibody titres increased with age, consistent with our observations (Stanescu et al., 2025). Among HCWs, studies from Slovakia and Croatia found seropositivity rates of 85.7% and 92.3%, respectively, with increased susceptibility in the 30–39-year cohort in Slovakia (2018) and antibody titres increasing with age (Urbančíková et al., 2026; Zember et al., 2024). Our results align with these studies, highlighting the influence of age, vaccination policy, and cohort effects on measles immunity and the persistence of immunity gaps in both the general population and among HCWs.

In Hungary, only sporadic measles cases (<5 per year) were reported between 2010 and 2017. However, an outbreak involving 54 clinically suspected cases occurred in Makó and Szeged (Csongrád-Csanád County, Souteast Hungary) between 29 January and 10 March 2017. Laboratory confirmation was obtained for 15 cases, including 13 healthcare workers in a university clinic. Sequencing identified epidemiological links with Romanian and Italian cases (Lengyel et al., 2018). Later in July 2017, another laboratory-confirmed cluster occurred in Nyíregyháza (Szabolcs-Szatmár-Bereg County, Northeast Hungary), involving six unvaccinated Romanian children hospitalized with measles and nosocomial transmission to two Hungarian HCWs (Emberi Erőforrások Minisztériuma, 2017). In total, 14 suspected measles cases were reported in 2018 and 23 in 2019. Since 2020, only one sporadic case was reported in 2023, while 18 cases occurred in 2024 (National Center for public health and pharmacy, 2024b; https://www.antsz.hu/felso_menu/temaink/jarvany/Fertozo_betegsegek/Fertozo_eves_jelentesk). These findings emphasize the importance of evaluating immunity, particularly given the resurgence of measles in Europe and documented nosocomial transmission in healthcare settings (Georgakopoulou et al., 2018; Angelo et al., 2019; Baccolini et al., 2020; Deal et al., 2021).

Changes in the Hungarian vaccination program – including vaccine type, number of doses, and age of administration - allowed comparison of antibody levels across different vaccination histories. Our findings suggest that different vaccine formulations may result different antibody levels but the time since the vaccine was administered may also be an influencing factor. The highest antibody titres were observed in individuals receiving the MMR-II and MMR-Priorix booster combination. High titres were also detected among individuals vaccinated once with the Leningrad-16 strain. Between 1969 and 1973, vaccination was introduced as a campaign and was not mandatory; therefore, not all individuals born in this period were vaccinated, and some were may also naturally immunized through infection or reinfection. Additionally, a measles outbreak occurred in Hungary during 1988–1989, with Pécs particularly affected (Rigó et al., 2020), and many cases occurred among individuals aged 16–22 years. According to public health regulations issued in 1989 (A szociális- és egészségügyi miniszter, 2026; https://research.physcon.uni-obuda.hu/VML/VML1989.pdf), individuals born between 1969 and 1973 who had contact with confirmed measles cases were revaccinated regardless of prior vaccination status. Consequently, most individuals in this cohort likely received at least one vaccine dose and a substantial proportion may also have been exposed to wild-type virus and/or revaccinated, which may explain the high antibody levels observed in this group.

Interestingly, unvaccinated individuals in our cohort showed the highest antibody titres, with mean and median values significantly exceeding those of vaccinated. This likely reflects the stronger and longer lasting (>55 years) immune memory induced by natural measles infection compared with vaccination, a phenomenon described previously (Christenson and Böttiger, 1994; Moss and Polack, 2001).

We also observed an association between age and measles susceptibility. Individuals aged 39–50 years (in 2024) showed the highest proportion of susceptibility. As this group represents a substantial portion of the workforce, ensuring adequate immunity in this cohort is particularly important. Increasing susceptibility was also observed among 19–35-year-old age groups. Serological testing can identify individuals requiring revaccination and may be cost-effective; the material cost of our screening was approximately €1.8 per individual.

Beyond vaccination policy implications, our findings highlight the value of targeted revaccination strategies. Revaccination may be considered for HCWs with low antibody titres, particularly those vaccinated with older formulations. Based on our serological results, individuals with antibody levels below 200 mIU/mL were offered MMR revaccination.

This study has several limitations: The sex and age distribution of participants was unequal. Screening included only those healthcare workers who were unable or unwilling to present vaccination records. Although this may affect representativeness, the large sample size (2541 of 5003 HCWs) still provides robust estimates, and loss of vaccination card is likely random rather than systematically associated with serostatus. Vaccination categories and immunization status were inferred from year of birth because individual vaccination records were unavailable. Even though the national vaccination coverage has historically exceeded 99%, misclassification cannot be completely excluded; some individuals may have been vaccinated later in life or exposed to wild-type measles virus, potentially influencing antibody levels and partially confounding comparisons between immunization groups. Migration-related differences in vaccination status likely had minimal impact on the results, as this was extremely rare (<5 individuals) in the studied population. Waning immunity may also lead to declining measles IgG titres over time (Yang et al., 2020), which could influence our findings; however, in some cases higher antibody levels were observed after older vaccine formulations. Finally, cell-mediated immune responses also contribute to measles immunity but were not evaluated in this study.

## 5 Conclusions

Our study highlights the variable immunogenicity of different measles vaccine formulations and emphasizes the importance of immunity screening for healthcare workers. Maintaining high vaccination coverage and protection level is essential in preventing measles outbreaks in healthcare settings. Taken together with international studies, these findings indicate that waning immunity or incomplete protection following earlier vaccination campaigns continues to be a common phenomenon across Europe.

## CRediT authorship contribution statement

**Bálint Timmer:** Writing – review & editing, Writing – original draft, Visualization, Software, Methodology, Investigation, Formal analysis, Data curation. **Benigna Balázs:** Writing – review & editing, Investigation, Data curation. **Ákos Boros:** Writing – review & editing, Validation, Methodology, Investigation. **Antal Tibold:** Writing – review & editing, Project administration, Investigation, Conceptualization. **Andor Sebestyén:** Writing – review & editing, Supervision, Resources, Funding acquisition, Conceptualization. **Gábor Reuter:** Writing – review & editing, Writing – original draft, Validation, Supervision, Methodology, Investigation, Formal analysis, Data curation, Conceptualization.

## Informed consent statement

All healthcare workers participating in the measles serostatus study received information in accordance with Hungarian legislation (Article 23/B (related to the measles serosurvey campaign among HCWs) of the NM Decree 18/1998).

## Institutional review board statement

The health data collection authorization number is KK/3940–1/2024 (University of Pécs). Approved on 12/02/2024.

## Funding

This research did not receive any specific grant from funding agencies in the public, commercial, or not-for-profit sectors.

## Declaration of competing interest

The authors declare that they have no known competing interests or personal relationships that have appeared to influence the work reported in this paper.

## Data Availability

Data will be made available on request.
