# Supplementary material for: Measles serostatus among health care workers in a university clinic in Hungary in 2024
Source: Prev Med Rep. 2026 May 8;66:103496. doi: 10.1016/j.pmedr.2026.103496 (PMC13188129; doi:10.1016/j.pmedr.2026.103496)

**Supplementary material**


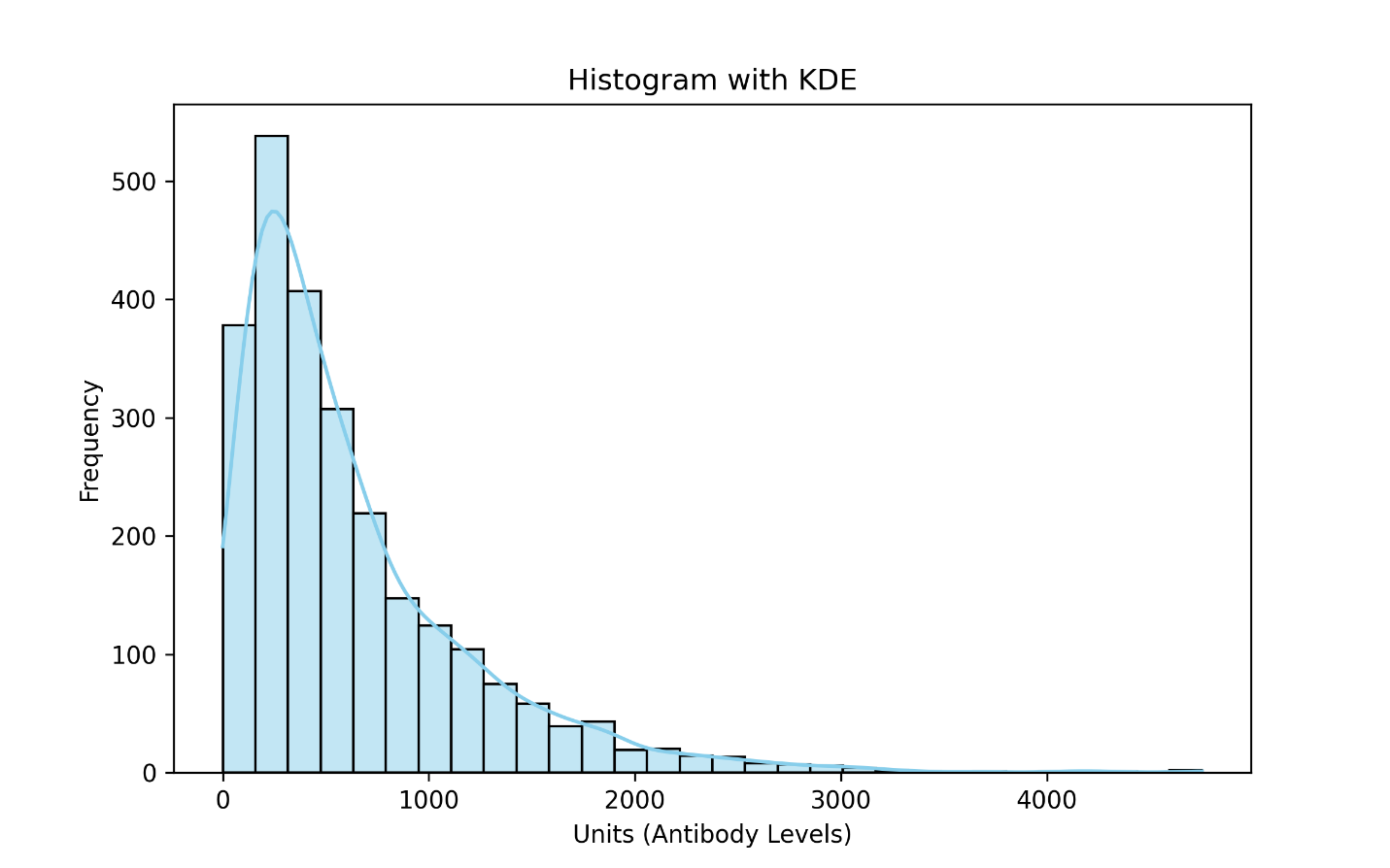


**Supplementary Figure 1.** Histogram shows that dataset of serum measles IgG titer (mIU/ml) is not normally distributed among healthcare workers (HCWs) in a Hungarian university clinic in 2024. KDE = Kernel density estimation


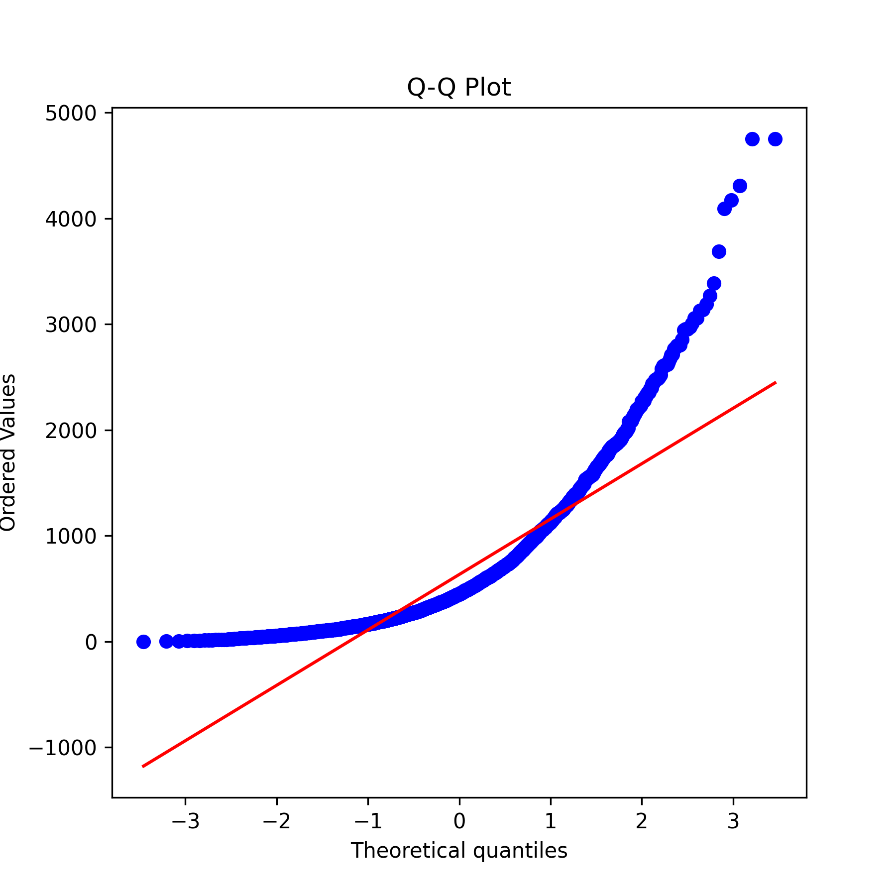


**Supplementary Figure 2.** QQ plot shows the dataset of serum measles IgG antibody titer (mIU/ml) is not normally distributed among healthcare workers (HCWs) in a Hungarian university clinic in 2024.

**
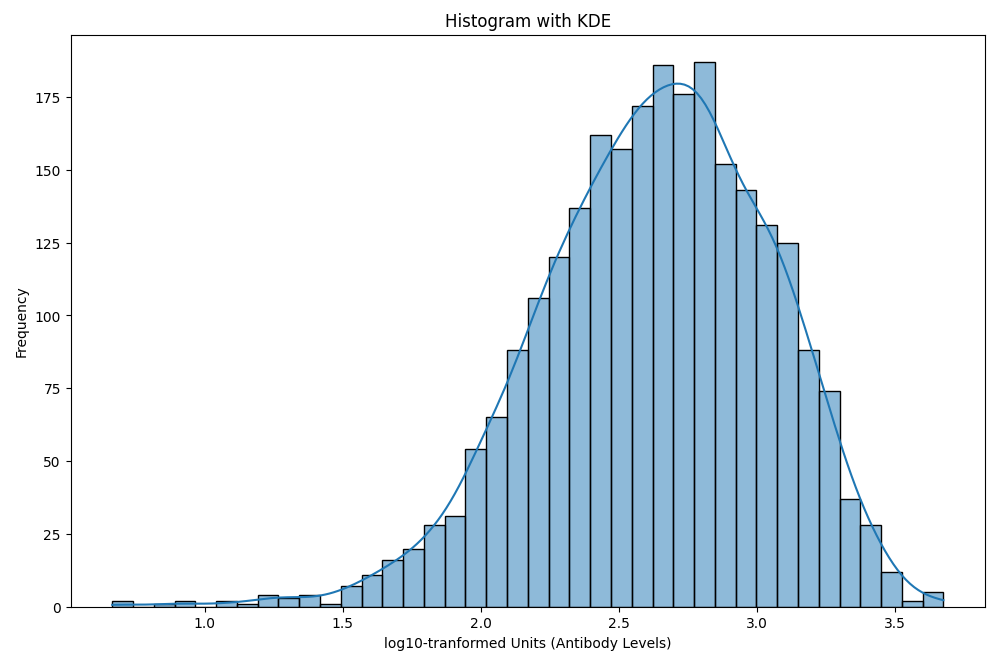
**

**Supplementary Figure 3.** Histogram shows that the dataset of log_10_ transformed serum measles IgG titer (mIU/ml) values is not normally distributed among healthcare workers (HCWs) in a Hungarian university clinic in 2024. KDE = Kernel density estimation


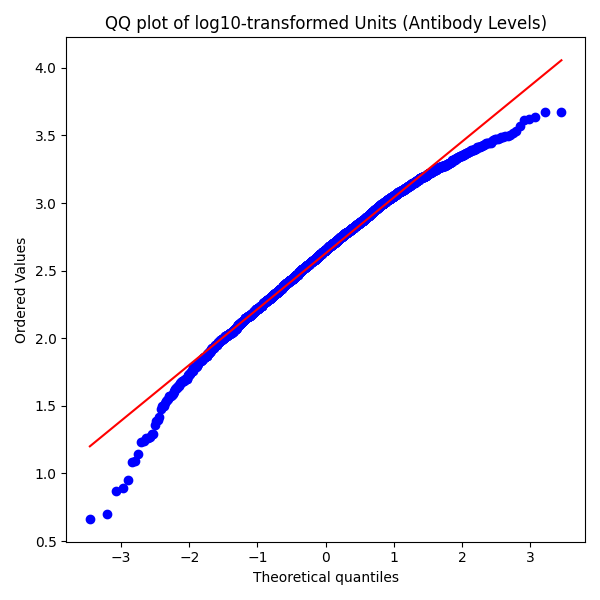


**Supplementary Figure 4.** QQ plot shows the dataset of log_10_ transformed serum measles IgG antibody titer (mIU/ml) values is not normally distributed among healthcare workers (HCWs) in a Hungarian university clinic in 2024.


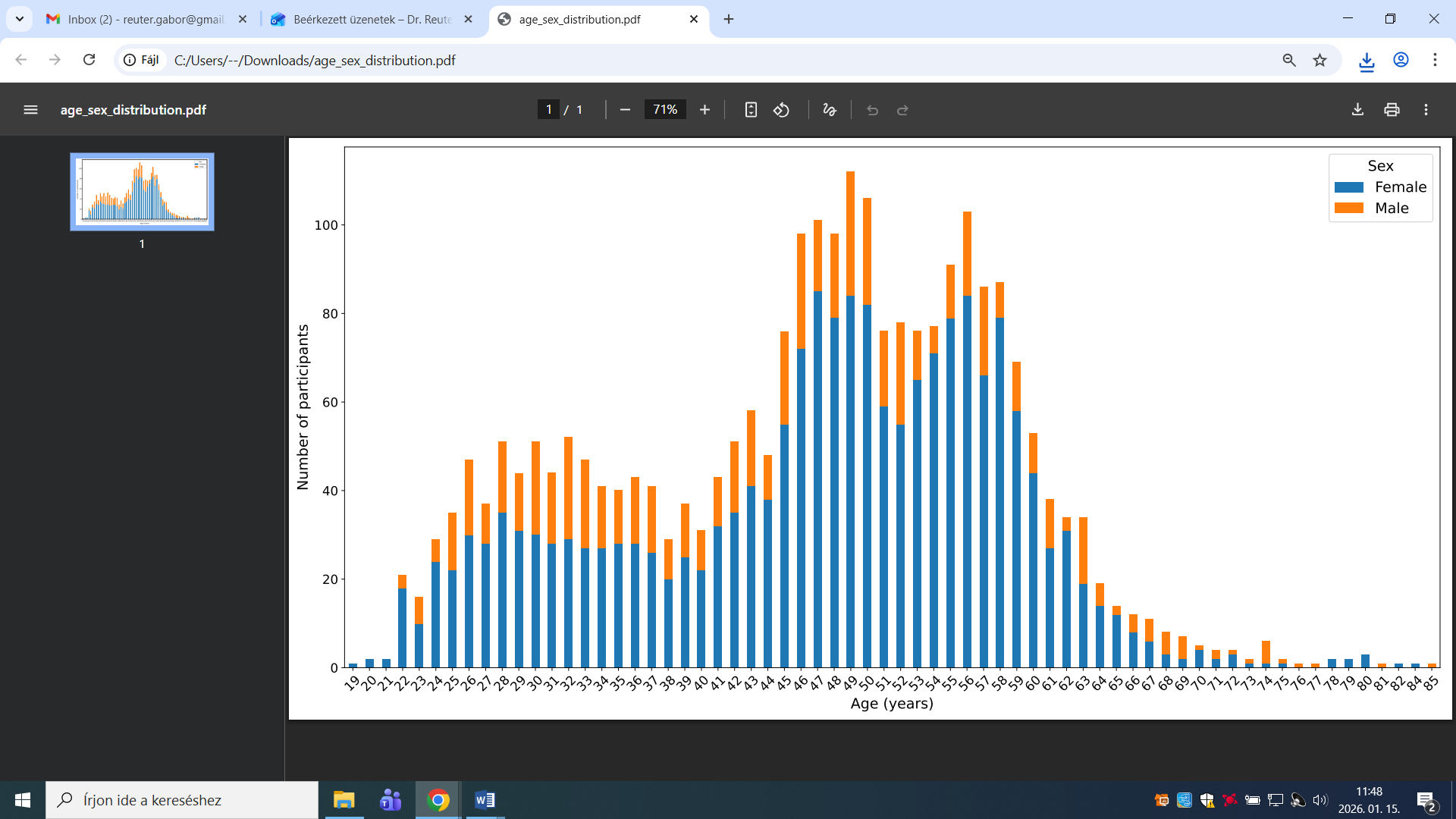


**Supplementary Figure 5.** Age (x-axis) and sex distributions of the HCW participants (N=2,541of the 5,003) among healthcare workers (HCWs) in a Hungarian university clinic in 2024.

**Supplementary Table 1.** Measles vaccination schedules in Hungary since 1968 according to the year of birth of participating healthcare workers. MMR = mumps-measles-rubella

| Vaccine(s) type | Year of birth | Number of participants |
| --- | --- | --- |
| no vaccination | <1968 | 508 |
| Leningrad-16 1X (Campaign) | 1968-1973 | 502 |
| Leningrad-16 2X | 1974-1977 | 417 |
| Leningrad-16-MR | 1978-1979 | 174 |
| Leningrad-16-Rimevax | 1980-1987 | 338 |
| Leningrad-16-MMR-II | 1988 | 43 |
| MR-MMR-II | 1989-1990 | 81 |
| MMR-Pluserix-MMR-II | 1991-1994 | 194 |
| MMR-Pluserix-Priorix | 1995 | 44 |
| MMR-II-Priorix | 1996-2004 | 239 |
| Priorix-Priorix | 2005- | 1 |

**Supplementary Table 2.** p-values after Post-hoc Dunn’s test for identification of differences in measles IgG antibody levels between specific vaccine groups among healthcare workers (HCWs) in a Hungarian university clinic in 2024.

|  | Leningrad-16-MMR-II | Leningrad-16-MR | Leningrad-16-Rimevax | Leningrad-16_1X (Campaign) | Leningrad-16_2X | MMR-II-Priorix | MMR-Pluserix-MMR-II | MMR-Pluserix-Priorix | MR-MMR-II | no vaccination |
| --- | --- | --- | --- | --- | --- | --- | --- | --- | --- | --- |
| Leningrad-16-MMR-II | 1 | 1 | 1 | 0.80 | 0.22 | 1 | 1 | 1 | 1 | 9.09E-07 |
| Leningrad-16-MR | 1 | 1 | 1 | 5.22E-12 | 1 | 0.06E-2 | 0.11 | 1 | 1 | 2.31E-41 |
| Leningrad-16-Rimevax | 1 | 1 | 1 | 6.43E-13 | 0.16 | 0.58E-2 | 0.80 | 1 | 1 | 2.13E-53 |
| Leningrad-16_1X (Campaign) | 0.80 | 5.22E-12 | 6.43E-13 | 1 | 4.09E-27 | 0.1 | 0.08E-2 | 0.07E-2 | 0.04E-2 | 3.07E-17 |
| Leningrad-16_2X | 0.22 | 1 | 0.16 | 4.09E-27 | 1 | 1.04E-08 | 0.03E-2 | 1 | 1 | 8.56E-84 |
| MMR-II-Priorix | 1 | 0.06E-2 | 0.58E-2 | 0.1 | 1.04E-08 | 1 | 1 | 0.18 | 0.47 | 5.31E-22 |
| MMR-Pluserix-MMR-II | 1 | 0.11 | 0.80 | 0.08E-2 | 0.03E-2 | 1 | 1 | 1 | 1 | 1.28E-25 |
| MMR-Pluserix-Priorix | 1 | 1 | 1 | 0.07E-2 | 1 | 0.18 | 1 | 1 | 1 | 3.12E-13 |
| MR-MMR-II | 1 | 1 | 1 | 0.04E-2 | 1 | 0.47 | 1 | 1 | 1 | 8.42E-18 |
| no vaccination | 9.09E-07 | 2.31E-41 | 2.13E-53 | 3.07E-17 | 8.56E-84 | 5.31E-22 | 1.28E-25 | 3.12E-13 | 8.42E-18 | 1 |

Supplementary data:

1. Representativity:

The total workforce of the tested healthcare facilities in a Hungarian university clinic consists of 5,003 individuals, of which we tested 2,541 in 2024. With the confidence level of 95% (Z-score = 1.96, margin of error 5%):

n= [(1.96)^2^⋅0.5⋅(1−0.5)]/ (0.05)^2^​=0.9604/0.0025​=384.16

Out of 5,003 workers at least 384 needs to be tested for a representative study hence our dataset is representative with 5% margin of error.

With the confidence level of 95% (Z-score = 1.96, margin of error 2%):

N=[(1.96)^2^⋅0.5⋅(1−0.5)]/ (0.02)^2^​=0.9604/0.0004​=2,401

Out of 5,003 workers at least 2,401 needs to be tested for a representative study hence our dataset is representative with 2% margin of error.

1. Sex Distribution:

Female: 1899 (74.73%)

Male: 642 (25.27%)

1. Statistics for Males (M)

Units (Antibody Levels, mIU/ml):

Min: 4.62

Max: 4309.35

Median: 423.72

Mean: 608.58

Q1: 218.94

Q3: 792.15

Age (year):

Min: 22

Max: 85

Median: 45

Mean: 44.64

Q1: 33

Q3: 53

Unit Range Distribution:

>200mIU/ml: 493 (76.79%)

<150mIU/ml: 96 (14.95%)

150-200mIU/ml: 53 (8.26%)

Immunisation Status (>200 mIU/ml):

Yes: 493 (76.79%)

No: 149 (23.21%)

1. Statistics for Females (F)

Units (Antibody Levels, mIU/ml):

Min: 0.0

Max: 4751.29

Median: 454.92

Mean: 640.87

Q1: 230.90

Q3: 860.29

Age (year):

Min: 19

Max: 84

Median: 49

Mean: 47

Q1: 40

Q3: 55

Unit Range Distribution:

>200 mIU/ml: 1510 (79.51%)

<150 mIU/ml: 255 (13.43%)

150-200 mIU/ml: 134 (7.06%)

Immunisation Status (>200 mIU/ml):

Yes: 1510 (79.51%)

No: 389 (20.49%)

1. Overall statistics:

Age (year):

Minimum: 19

Maximum: 85

Median: 48

Mean: 46

Q1: 37

Q3: 55

Measles IgG antibody levels (mIU/ml):

Min: 0.0

Max: 4751.28

Median: 448.57

Mean: 632.71

Q1: 227.19

Q3: 844.43

Antibody level categories:

>200 mIU/ml: 2003 (78.83%)

<150 mIU/ml: 351 (13.81%)

150-200 mIU/ml: 187 (7.36%)

Immunisation status (>200 mIU/ml):

Yes: 2003 (78.83%)

No: 538 (21.17%)

1. Statistics for Vaccine Types:

Statistics for Vaccine Type: Leningrad-16/Rimevax (n=338)

Units (Antibody Levels, mIU/ml):

Min: 4.62

Max: 4309.35

Median: 349.6

Mean: 442.22

Q1: 185.26

Q3: 573.59

Unit Range Distribution:

>200 mIU/ml: 244 (72.19%)

<150 mIU/ml: 66 (19.53%)

150-200 mIU/ml: 28 (8.28%)

Immunisation Status (>200 mIU/ml):

Y: 244 (72.19%)

N: 94 (27.81%)

==================================================

Statistics for Vaccine Type: MMR-II/Priorix (n=240)

Units (Antibody Levels, mIU/ml):

Min: 47.07

Max: 2011.0

Median: 463.92

Mean: 532.18

Q1: 275.01

Q3: 683.02

Unit Range Distribution:

>200 mIU/ml: 207 (86.25%)

<150 mIU/ml: 22 (9.17%)

150-200 mIU/ml: 11 (4.58%)

Immunisation Status (>200 mIU/ml):

Yes: 207 (86.25%)

No: 33 (13.75%)

==================================================

Statistics for Vaccine Type: MMR-Pluserix/Priorix (n=44)

Units (Antibody Levels, mIU/ml):

Min: 24.79

Max: 1005.71

Median: 290.42

Mean: 356.39

Q1: 191.01

Q3: 491.49

Unit Range Distribution:

>200 mIU/ml: 31 (70.45%)

150-200 mIU/ml: 7 (15.91%)

<150 mIU/ml: 6 (13.64%)

Immunisation Status (>200 mIU/ml):

Yes: 31 (70.45%)

No: 13 (29.55%)

==================================================

Statistics for Vaccine Type: MMR-Pluserix/MMR-II (n=194)

Units (Antibody Levels, mIU/ml):

Min: 31.4

Max: 1733.14

Median: 374.02

Mean: 490.21

Q1: 250.77

Q3: 615.53

Unit Range Distribution:

>200 mIU/ml: 161 (83.00%)

<150 mIU/ml: 21 (10.82%)

150-200 mIU/ml: 12 (6.18%)

Immunisation Status (>200 mIU/ml):

Yes: 161 (83.00%)

No: 33 (17.00%)

==================================================

Statistics for Vaccine Type: MR/MMR-II (n=81)

Units (Antibody Levels, mIU/ml):

Min: 61.15

Max: 1890.78

Median: 348.13

Mean: 417.41

Q1: 201.03

Q3: 571.83

Unit Range Distribution:

>200 mIU/ml: 62 (76.54%)

<150 mIU/ml: 11 (13.58%)

150-200 mIU/ml: 8 (9.88%)

Immunisation Status (>200 mIU/ml):

Yes: 62 (76.54%)

No: 19 (23.46%)

==================================================

Statistics for Vaccine Type: Leningrad-16/MMR-II (n=43)

Units (Antibody Levels, mIU/ml):

Min: 76.17

Max: 1167.53

Median: 394.52

Mean: 478.75

Q1: 256.89

Q3: 614.35

Unit Range Distribution:

>200 mIU/ml: 38 (88.37%)

<150 mIU/ml: 4 (9.30%)

150-200 mIU/ml: 1 (2.33%)

Immunisation Status (>200 mIU/ml):

Yes: 38 (88.37%)

No: 5 (11.63%)

==================================================

Statistics for Vaccine Type: Leningrad-16/MR (n=174)

Units (Antibody Levels, mIU/ml):

Min: 22.95

Max: 2210.46

Median: 286.93

Mean: 394.39

Q1: 168.83

Q3: 503.47

Unit Range Distribution:

>200 mIU/ml: 116 (66.67%)

<150 mIU/ml: 33 (18.96%)

150-200 mIU/ml: 25 (14.37%)

Immunisation Status (>200 mIU/ml):

Yes: 116 (66.67%)

No: 58 (33.33%)

==================================================

Statistics for Vaccine Type: Leningrad-16_2X (n=417)

Units (Antibody Levels, mIU/ml):

Min: 13.97

Max: 3007.16

Median: 260.29

Mean: 386.4

Q1: 143.65

Q3: 484.34

Unit Range Distribution:

>200 mIU/ml: 255 (61.15%)

<150 mIU/ml: 113 (27.10%)

150-200 mIU/ml: 49 (11.75%)

Immunisation Status (>200 mIU/ml):

Yes: 255 (61.15%)

No: 162 (38.85%)

==================================================

Statistics for Vaccine Type: Leningrad-16_1X (Campaign) (n=502)

Units (Antibody Levels, mIU/ml):

Min: 5.04

Max: 2972.98

Median: 558.24

Mean: 690.54

Q1: 295.94

Q3: 983.43

Unit Range Distribution:

>200 mIU/ml: 426 (84.86%)

<150 mIU/ml: 40 (7.97%)

150-200 mIU/ml: 36 (7.17%)

Immunisation Status (>200 mIU/ml):

Yes: 426 (84.86%)

No: 76 (15.14%)

==================================================

Statistics for Vaccine Type: unvaccinated (no vaccination) (n=508)

Units (Antibody Levels, mIU/ml):

Min: 0.0

Max: 4751.29

Median: 1074.58

Mean: 1159.32

Q1: 557.63

Q3: 1581.33

Unit Range Distribution:

>200 mIU/ml: 463 (91.14%)

<150 mIU/ml: 35 (6.89%)

150-200 mIU/ml: 10 (1.97%)

Immunisation Status (>200 mIU/ml):

Yes: 463 (91.14%)

No: 45 (8.86%)

==================================================

1. Results of normality tests:

Non-transformed serum anti-measles IgG antibody titres:

Shapiro-Wilk test results: Statistic=0.81, p-value<0.001

Conclusion: The data does not appear to be normally distributed (p<0.05).

Log_10_ transformed serum anti-measles IgG antibody titres:

Shapiro–Wilk: p = 1.76E-07

D’Agostino–Pearson: p = 1.45E-24

Anderson–Darling statistic: 4.26

Anderson–Darling critical values: 1.09

Conclusion: The data does not appear to be normally distributed, different statistical tests uniformly rejecting normality.

1. Results of Kruskal-Wallis comparisons

Between vaccinated and unvaccinated (no vaccination) groups excluding Leningrad-16 1X (Campaign) group:

p=8.80E-95

Between different vaccine combinations excluding Leningrad-16 1X (Campaign) group: p=1.63E-98

1. Differences in the proportion of susceptible individuals between groups of different vaccination status


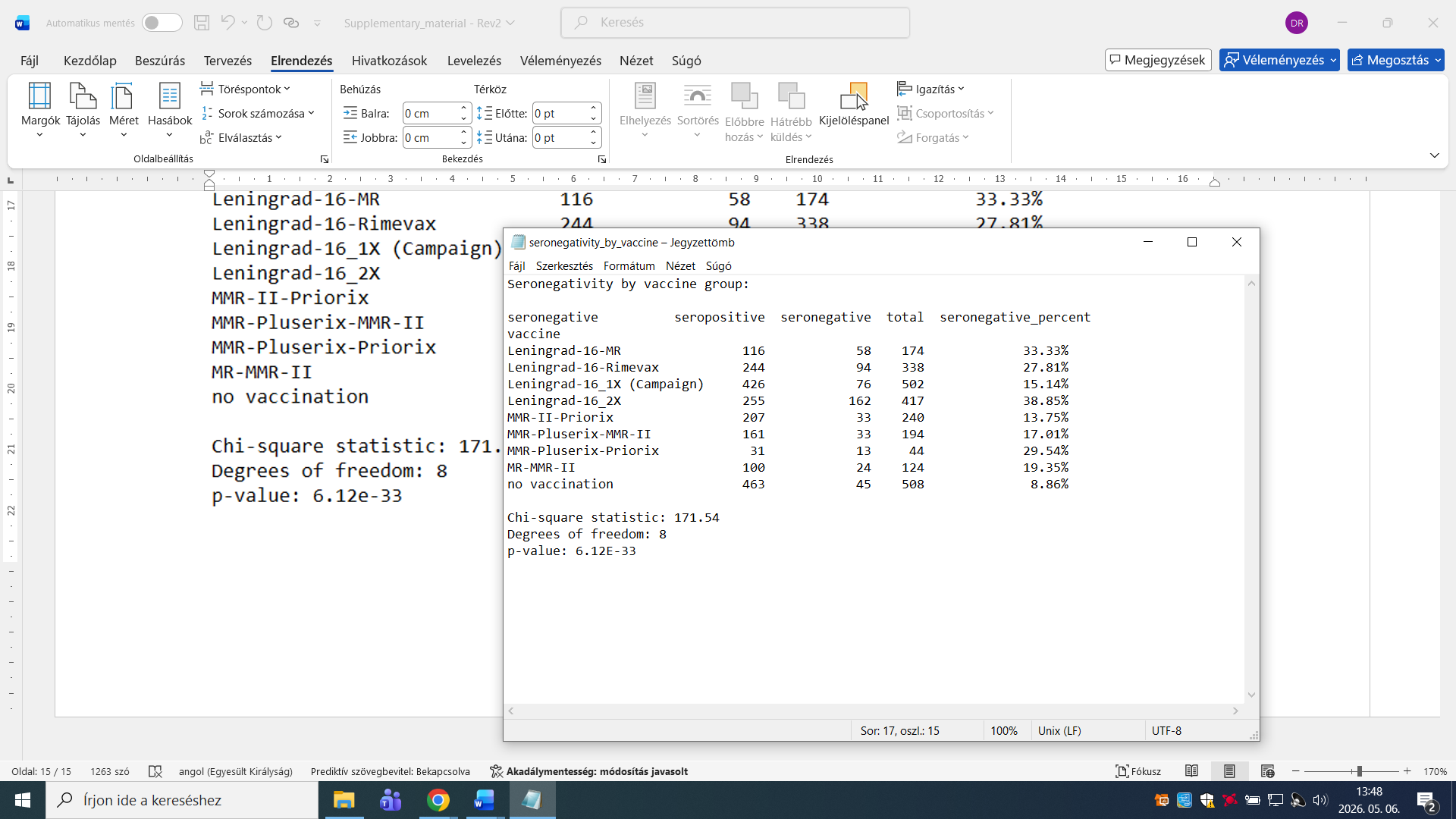

Supplement: Supplementary file 1 — Supplementary material [file mmc1.docx]
